# Clinical features of acute-onset autoimmune hepatitis: a systematic review and meta-analysis

**DOI:** 10.1016/j.clinsp.2026.101118

**Published:** 2026-09-24

**Authors:** Xiaotian Dong, Jinhua Zhang, Yufan Guan, Jiaze An

**Affiliations:** a Joint Logistic Support Force 970th Hospital, Yantai, China; b Department of Hepatobiliary Surgery, The Fourth Military Medical University, China

**Keywords:** Autoimmune hepatitis, Acute-onset autoimmune hepatitis, Auto-immune disease, Clinical feature, Meta-analysis

## Abstract

**Background and aims:**

Autoimmune Hepatitis (AIH), a rare chronic progressive inflammatory disease of the liver, and acute-onset AIH usually lack typical manifestations and have a poor prognosis. Hence, we have systematically examined all accessible data on the clinical features of acute-onset AIH to aid clinicians in deepening their understanding of the disease.

**Methods:**

Eligible studies were gathered from a computer search of literature published from the inception of the database to June 2024 in PubMed and CNKI. Data on basic features, clinical manifestations, laboratory examinations, clinical outcomes, and pathological results were collected as relevant outcomes.

**Results:**

Thirty-two retrospective studies encompassing 2,561 patients, and four cohort studies involving 419 patients were ultimately included. Approximately 31% of AIH patients present with acute disease onset, with 58% being acute non-severe, 25% acute severe, and 17% commencing with fulminant liver failure. Patients with acute-onset AIH tend to be younger (OR = 0.25, 95% CI: -0.449 to -0.057) and exhibit a diminishing female predilection that increases with severity compared to the more chronic classic AIH (OR = 0.65, 95% CI: 0.569‒0.727). The presence of comorbid extrahepatic autoimmune disease showed a potential protective effect when compared with C-AIH (OR = 0.63, 95% CI: 0.455‒0.874).

**Conclusions:**

About 30% of patients with AIH exhibit an acute disease onset, understanding its proportion and clinical features aids clinicians in making timely diagnoses to prevent severe consequences. However, in existing studies, the results of laboratory tests and histologic examinations of A-AIH are influenced by AC-AIH to varying degrees, which somewhat restricts the clinicians' understanding.

## Highlights

• Systematic review of acute-onset autoimmune hepatitis.
• Identifies key predictors of severity: bilirubin, INR, and extrahepatic autoimmunity.
• Acute vs. chronic AIH differ in symptoms, lab findings, and outcomes.
• Anorexia and jaundice are key symptoms distinguishing acute AIH.

## Introduction

Autoimmune Hepatitis (AIH) is a chronic liver disease, driven by abnormal autoimmunity, likely precipitated by the interplay of genetic susceptibility, environmental factors, and triggering events.^1^ The clinical presentation of AIH varies greatly, it typically has a chronic onset, and most of its symptoms are nonspecific, making physical examinations rather uninformative.

To address the need for reliable, user-friendly diagnostic tools in clinical practice, the International Autoimmune Hepatitis Group (IAIHG) introduced diagnostic criteria in 1993, which were later revised in 1999 The comprehensive diagnostic scoring system,^2^ though highly sensitive and specific in AIH diagnosis, is complicated and challenging to widely implement. Hence, the IAIHG proposed a simplified scoring system in 2008.^2^ Despite the absence of specific biomarkers, the current diagnostic scoring system is quite effective and clinically applicable.^3^

However, approximately 20% of patients exhibit acute symptoms,^4^ presenting clinical features that are more elusive. These individuals could be experiencing true acute AIH or Acute on Chronic AIH (AC-AIH), typically manifesting as acute jaundice and/or coagulation dysfunction. Based on INR and encephalopathy, these cases can be further classified as Acute AIH (A-AIH), Acute Severe AIH (AS-AIH), or fulminant AIH (ALF-AIH).^5^ Notably, patients with an acute AIH presentation differ significantly from those with classic, Chronic Autoimmune Hepatitis (C-AIH) in terms of histomorphology, serology, and laboratory characteristics.^6^

Due to these variable clinical features, scoring systems often fall short in diagnosing acute AIH, making early diagnosis and differential diagnosis quite challenging.^4^^,^^5^ Acute-onset AIH's complexity, diagnostic difficulty, rapid progression, and poor prognosis warrant thorough examination and summarization. Our study is the first to systematically review the clinical features of acute AIH, encompassing clinical manifestations, outcomes, serologic and laboratory tests, and histologic examinations. The aim is to enhance clinicians' understanding of acute AIH and thereby improve their diagnostic and therapeutic capabilities.

## Method

### Literature search strategy

We conducted a comprehensive search on both PubMed and CNKI (China National Knowledge Infrastructure), covering the time frame from the establishment of these databases up to May 30, 2024. We utilized the search terms 'acute autoimmune hepatitis', along with the combinations of 'Autoimmune hepatitis' AND 'acute', 'acute onset', 'severe', and 'fulminant hepatic failure'. Furthermore, we combed through the reference lists of relevant articles to identify any additional suitable studies.

### Study selection and data extraction

Our study selection encompassed articles that specifically explored acute-onset AIH. We also included studies pertaining to AIH, if they separately grouped clinical features of acute AIH and had a sample size of 10 or more patients. Our systematic reviews and meta-analyses conform to the protocols developed by authors XTD, JHZ, and YFG and the Preferred Reporting Items for Systematic Reviews and Meta-Analyses (PRISMA) guidelines. Studies available despite searching all literature in relevant databases were retrospective studies.

Two independent researchers, Xiaotian Dong & Jinhua Zhang, undertook the data extraction process. The extracted data included the first author's name, year of publication, study design, study region, diagnostic criteria, basic features, clinical manifestations, laboratory examinations, clinical outcomes, and pathological results. In instances of discrepancy, the two researchers would re-examine the data and negotiate to reach an agreement. If necessary, expert Jiaze An was consulted to ensure a consensus among all investigators.

### Assessment of the study quality and risk of bias

The quality of each study was assessed independently by two reviewers. To determine the potential occurrence of publication bias in randomized studies, assessment tools from the Cochrane Collaboration were utilized. Evaluations were performed in various domains, with studies classified as possessing either ‘low risk”, “unclear risk”, or “high risk” of bias. For observational studies, the evaluation of bias was conducted using the modified Newcastle-Ottawa Scale (NOS). This scale considers three aspects: participant selection (up to a maximum of 4-points), comparability (up to a maximum of 2-points), and the exposure or outcomes of study participants (up to a maximum of 3-points). Based on the overall scores obtained, studies were categorized as either high quality (≥ 7), fair quality (4–6), or low quality (≤ 4).

### Statistical analysis

Review Manager Version 5.3 software was employed for statistical data analysis. We computed pooled proportion, pooled mean, mean difference, odds ratios and 95% Confidence Intervals to examine various aspects of acute-onset autoimmune hepatitis patients, using a random-effects model. Heterogeneity was assessed via the χ^2^ test (α = 0.1) and was quantitatively measured by *I*^2^. High heterogeneity was considered if (p < 0.1, *I*^2^ > 50%). We assessed potential publication bias using the Egger's regression model.

## Result

In summary, our database search yielded 3,920 articles. After eliminating duplicates, 149 records were assessed at the title/abstract stage, and 126 were evaluated at the full-text stage. Our PRISMA flowchart (Supplementary Fig. 1) provides detailed explanations for the exclusion of 90 studies at the full-text stage. Eventually, 36 studies^4^, ^5^, ^6^, ^7^, ^8^, ^9^, ^10^, ^11^, ^12^, ^13^, ^14^, ^15^, ^16^, ^17^, ^18^, ^19^, ^20^, ^21^, ^22^, ^23^, ^24^, ^25^, ^26^, ^27^, ^28^, ^29^, ^30^, ^31^, ^32^, ^33^, ^34^, ^35^, ^36^, ^37^, ^38^, ^39^ were included in this systematic review and quantitative synthesis (Supplementary Table 1).

Supplementary Table 1 encapsulates our findings. The majority of the studies (24 out of 36) were conducted in Asia, with Japan leading with 16 studies,^6^^,^^8^, ^9^, ^10^, ^11^^,^^13^^,^^16^, ^17^, ^18^^,^^20^^,^^23^^,^^24^^,^^26^^,^^29^^,^^30^^,^^38^ followed by six studies^31^, ^32^, ^33^, ^34^^,^^37^^,^^39^ from China, and one study each from Saudi Arabia^5^ and India.^36^ The vast majority of the research (33 out of 36 studies) diagnosed AIH using the 1999 original criteria^40^ and/or the 2008 simplified criteria,^2^ two studies^15^^,^^28^ conducted prior to 1999 utilized published criteria,^41^ and one study^25^ focused on children used the classical criteria for diagnosing AIH in children.^42^

Acute onset was defined as the clinical presentation of acute hepatitis with acute-onset liver injury, with onset ranging from less than one month to less than a year. All studies excluded other diseases affecting the liver, such as hepatitis B and overlap syndrome. The definition of AS-AIH fell into two main groups, 1) INR ≥1.5 without encephalopathy; 2) Prothrombin Time (PT) activity < 50% of control or total bilirubin level more than 20 mg/dL. ALF-AIH was primarily defined by the presence of hepatic encephalopathy, accompanied by abnormalities in INR or PT. As the definitions of acute non-severe AIH and ALF-AIH were relatively consistent, we did not further classify AS-AIH.

### Characteristic

Of all patients diagnosed with AIH, 31.4% presented with an acute onset. Among those experiencing acute manifestations of AIH, 58.4% were acutely non-severe, 25.3% were acutely severe, and 16.9% initiated with fulminant liver failure (Table 1).

**Table 1:** Number of cases of acute-onset AIH among all AIH cases and percentage of each severity level in acute-onset AIH.

	Studies (n)	Participants (n)	Heterogeneity		Model	Meta analysis		Egger
			p	*I*^2^		Pooled proportion	95% CI	
**Acute-onset AIH/ AIH**	5	527	0.050	57.786	Random	0.314	0.255‒0.380	0.94
**A-AIH / Acute onset AIH**	3	208	0.341	7.065	Random	0.584	0.511‒0.653	0.29
**AS-AIH / Acute onset AIH**	4	309	0.980	0.000	Random	0.253	0.207‒0.304	0.96
**ALF-AIH / Acute onset AIH**	3	208	0.114	53.934	Random	0.169	0.102‒0.268	0.51

Within the A-AIH, AS-AIH, and ALF-AIH groups, the mean age was 49.9 (95% CI: 44.578‒55.204), 46.2 (95% CI: 42.934‒49.400), and 50.3 (95% CI: 42.453‒58.203), respectively, and these differences were not statistically significant (Table 2).

**Table 2:** Basic characteristics of patients with acute-onset AIH.

	Studies (n)	Participants (n)	Heterogeneity		Model	Meta analysis		Egger
			p	*I*^2^		Pooled mean/ Proportion	95% CI	
**A-AIH**								
Age	19	612	0.000	98.816	Random	49.891	44.578‒55.204	0.00
Sex (females)	24	1070	0.007	46.644	Random	0.810	0.770‒0.845	0.06
**AS-AIH**								
Age	7	357	0.057	50.940	Random	46.167	42.934‒49.400	0.06
Sex (females)	9	415	0.921	0.000	Random	0.743	0.698‒0.783	0.43
**ALF-AIH**								
Age	6	134	0.000	91.888	Random	50.328	42.453‒58.203	0.03
Sex (females)	6	134	0.227	25.332	Random	0.169	0.569‒0.727	0.10

In terms of gender distribution, the female proportion in the A-AIH, AS-AIH, and ALF-AIH groups was 81% (95% CI: 0.770‒0.845), 74% (95% CI: 0.698‒0.783), and 65% (95% CI: 0.569‒0.727), respectively. Unlike age, the female to male ratio tended to decrease with the severity of the condition, and a significant difference in the female ratio was observed between the A-AIH and ALF-AIH groups (Table 2).

### Symptoms

In the 36 papers we reviewed, 21 symptoms were reported. A meta-analysis was conducted on the 13 symptoms with two or more reports, namely jaundice, fatigue, abdominal pain, anorexia, bloating, ascites, oil aversion, nausea, weight loss, urine discoloration, lower limb swelling, fever, and arthralgia. The most commonly reported were jaundice 79% (95% CI: 0.656‒0.879), fatigue 65% (95% CI: 0.462‒0.794), anorexia 64% (95% CI: 0.514‒0.756), urine discoloration 44% (95% CI: 0.348‒0.544), and oil aversion 40% (95% CI: 0.312‒0.491). The least common were lower limb swelling 16% (95% CI: 0.061‒0.356) and arthralgia 6% (95% CI: 0.030‒0.105) (Table 3).

**Table 3:** Symptoms associated with acute-onset AIH.

	Studies (n)	Participants (n)	Heterogeneity		Model	Meta analysis		Egger
			p	*I*^2^		Pooled proportion	95% CI	
**ALL**								
Jaundice	9	265	0.001	69.993	Random	0.788	0.656‒0.879	0.08
Fatigue	7	231	0.000	79.143	Random	0.646	0.462‒0.794	0.01
Abdominal pain	4	167	0.084	54.958	Random	0.171	0.097‒0.285	0.01
Anorexia	5	149	0.087	50.719	Random	0.644	0.514‒0.756	0.14
Ventosity	3	105	0.904	0.000	Random	0.334	0.250‒0.429	0.45
Ascites	8	420	0.000	73.769	Random	0.287	0.189‒0.411	0.57
Detesting oil	4	113	0.952	0.000	Random	0.399	0.312‒0.491	0.25
Nausea	3	85	0.123	52.291	Random	0.251	0.137‒0.415	0.28
Weight loss	3	97	0.867	0.000	Random	0.176	0.112‒0.266	0.17
Yellow urine	3	97	0.628	0.000	Random	0.444	0.348‒0.544	0.67
Lower limb swelling	5	149	0.001	79.929	Random	0.159	0.061‒0.356	0.02
Fever	3	130	0.012	77.353	Random	0.301	0.155‒0.503	0.77
Arthralgia	4	167	0.828	0.000	Random	0.057	0.030‒0.105	0.01
Extrahepatic autoimmune disease	9	912	0.785	0.000	Random	0.216	0.191‒0.245	0.15
Thyroid disease	5	300	0.084	51.381	Random	0.163	0.104‒0.245	0.49
**A-AIH**								
Jaundice	7	225	0.003	69.390	Random	0.745	0.608‒0.846	0.38
Fatigue	6	215	0.000	78.287	Random	0.599	0.423‒0.752	0.04
Abdominal pain	4	167	0.084	54.958	Random	0.171	0.097‒0.285	0.02
Anorexia	4	133	0.285	20.777	Random	0.620	0.522‒0.709	0.68
Ventosity	3	105	0.904	0.000	Random	0.334	0.250‒0.429	0.45
Ascites	5	128	0.612	0.000	Random	0.282	0.210‒0.368	0.18
Detesting oil	3	97	0.894	0.000	Random	0.392	0.300‒0.492	0.46
Weight loss	3	97	0.867	0.000	Random	0.176	0.112‒0.266	0.17
Yellow urine	3	97	0.628	0.000	Random	0.444	0.348‒0.544	0.67
Lower limb swelling	4	133	0.000	83.617	Random	0.127	0.035‒0.370	0.02
Fever	2	106	0.003	88.350	Random	0.288	0.097‒0.604	‒
Arthralgia	4	167	0.828	0.000	Random	0.057	0.030‒0.105	0.01
Extrahepatic autoimmune disease	8	623	0.792	0.000	Random	0.225	0.194‒0.260	0.08
Thyroid disease	5	300	0.084	51.381	Random	0.163	0.104‒0.245	0.42
**AS-AIH**								
Ascites	3	292	0.000	91.478	Random	0.311	0.090‒0.672	0.73
Extrahepatic autoimmune disease	2	275	0.296	8.292	Random	0.207	0.158‒0.266	-

Between 19%‒24% of acute onset autoimmune hepatitis cases also presented with other autoimmune diseases, with no significant differences in prevalence among various severity levels. The most frequently reported comorbid condition was thyroid disease, occurring in 16.3% (95% CI: 0.104‒0.245) of A-AIH cases (Table 3).

### Laboratory examination

White Blood Cell (WBC) and Platelet (PLT) counts were most often included in the studies of A-AIH, reports of these counts in AS-AIH and ALF-AIH were not sufficient, and although the heterogeneity was high (*I*^2^ > 90), the combined results all fell within the normal range (Table 4).

**Table 4:** Laboratory examination results pertaining to acute-onset AIH.

	Studies (n)	Participants (n)	Heterogeneity			Meta analysis		Egger
			p	*I*^2^	Model	Pooled mean	95% CI	
**ALL**								
WBC (10^9^/L)	3	148	0.000	95.945	Random	5.466	3.809‒7.124	0.18
PLT (10^9^/L)	4	390	0.000	99.035	Random	164.482	86.501‒242.462	0.83
TB (mg/dL)	21	737	0.000	99.600	Random	12.109	7.424‒16.794	0.02
ALB (g/L)	10	274	0.000	92.818	Random	32.862	30.973‒34.750	0.53
GLB (g/L)	10	503	0.000	96.962	Random	30.667	27.952‒33.382	0.22
AST (IU/L)	15	734	0.000	99.702	Random	694.471	455.147‒933.796	0.01
ALT (IU/L)	23	952	0.000	99.397	Random	693.336	480.741‒905.932	0.19
ALP (IU/L)	12	444	0.000	97.548	Random	345.002	277.342‒412.661	0.00
GGT (IU/L)	5	150	0.000	80.384	Random	194.924	150.817‒239.031	0.86
PT (%)	8	340	0.000	97.943	Random	67.114	52.245‒81.982	0.73
INR	9	526	0.000	99.733	Random	2.168	1.527‒2.809	0.54
**A-AIH**								
WBC (10^9^/L)	3	119	0.000	93.748	Random	5.527	3.920‒7.135	0.96
PLT (10^9^/L)	3	119	0.000	98.759	Random	156.845	46.586‒267.103	0.24
TB (mg/dL)	15	223	0.000	93.180	Random	7.579	5.897‒9.261	0.49
ALB (g/L)	9	229	0.000	91.649	Random	33.432	31.469‒35.395	0.39
GLB (g/L)	7	144	0.000	96.136	Random	29.938	25.443‒34.433	0.43
AST (IU/L)	11	351	0.000	92.388	Random	682.292	584.030‒780.554	0.72
ALT (IU/L)	15	458	0.000	94.256	Random	724.323	563.935‒884.710	0.71
ALP (IU/L)	8	321	0.000	93.874	Random	403.649	315.836‒491.462	0.62
GGT (IU/L)	4	134	0.000	84.642	Random	192.798	134.610‒250.985	0.92
PT (%)	7	231	0.000	91.614	Random	81.945	74.585‒89.304	0.78
INR	4	138	0.000	96.154	Random	1.552	1.296‒1.809	-0.40
IgG (g/L)	17	496	0.000	97.431	Random	23.717	20.669‒26.765	0.70
**AS-AIH**								
TB (mg/dL)	5	300	0.090	50.278	Random	15.461	13.017‒17.905	0.73
AST (IU/L)	2	266	0.000	99.648	Random	795.622	‒334.580‒1925.825	-
ALT (IU/L)	6	324	0.000	98.567	Random	589.840	135.136‒1044.543	0.22
ALP (IU/L)	2	38	0.000	92.820	Random	307.908	120.073‒495.743	-
PT (%)	4	77	0.000	90.661	Random	43.416	38.367‒48.465	0.52
INR	2	266	0.127	57.116	Random	2.04	1.895‒2.184	-
IgG (g/L)	5	82	0.093	49.681	Random	22.101	19.282‒24.920	0.03
**ALF-AIH**								
TB (mg/dL)	6	125	0.003	72.519	Random	22.555	19.686‒25.425	0.19
GLB (g/L)	2	88	0.118	59.165	Random	34.727	32.578‒36.876	-
ALT (IU/L)	6	125	0.000	97.227	Random	726.800	299.574‒1154.026	0.20
ALP (IU/L)	4	113	0.000	95.715	Random	282.466	207.558‒357.374	0.20
PT (%)	3	32	0.314	13.748	Random	27.509	23.952‒31.065	0.07
INR	3	93	0.171	43.346	Random	3.263	2.935‒3.591	0.88
IgG (g/L)	5	58	0.000	88.465	Random	24.570	20.895‒28.246	0.13

From the combined results, aminotransferase levels offered minimal utility in gauging disease severity. Despite high heterogeneity (*I*^2^ > 90), these levels showed no statistical differences between A-AIH, AS-AIH, and ALF-AIH (Table 4).

Total Bilirubin (TB), International Normalized Ratio (INR), and Prothrombin Time (PT) served as significant indicators for diagnosing acute AIH and differentiating AIH severity. These measures varied significantly among groups, with TB mg/dL: A-AIH 7.6 (95% CI: 5.897‒9.261), AS-AIH 15.5 (95% CI: 13.017‒17.905), ALF-AIH 22.6 (95% CI: 19.686‒25.425); INR: A-AIH 1.6 (95% CI: 1.296‒1.809), AS-AIH 2.0 (95% CI: 1.895‒2.184), ALF-AIH 3.3 (95% CI: 2.935‒3.591); and PT(%): A-AIH 81.9 (95% CI: 74.585‒89.304), AS-AIH 43.4 (95% CI: 38.367‒48.465), ALF-AIH 27.5 (95% CI: 23.952‒31.065) (Table 4).

The combined results of Globulin (GLB) levels were 29.9 (g/L) for A-AIH and 34.7 (g/L) for ALF-AIH, but the difference between the two groups was not statistically significant. The combined Immunoglobulin G (IgG) levels for each AIH subtype were approximately 22‒24 (g/L), and the differences among the three groups were not statistically significant (Table 4).

The rates of autoantibody positivity were as follows: ANA 77.3% (95% CI: 0.729‒0.811), ASMA 41.1% (95% CI: 0.331‒0.495), AMA 10.5% (95% CI: 0.062‒0.171), and LKM-1 5.2% (95% CI: 0.038‒0.072). Moreover, there were no significant differences among the various severity levels (Table 5).

**Table 5:** Autoantibodies detected in acute-onset AIH.

	Studies (n)	Participants (n)	Heterogeneity			Meta analysis		Egger
			p	*I*^2^	Model	Pooled proportion	95% CI	
**ALL**								
ANA+	32	1541	0.000	63.668	Random	0.773	0.729‒0.811	0.00
SMA+	23	1260	0.000	84.481	Random	0.411	0.331‒0.495	0.48
AMA+	9	593	0.001	69.498	Random	0.105	0.062‒0.171	0.61
LKM1	16	1016	0.325	11.197	Random	0.052	0.038‒0.072	0.01
**A-AIH**								
ANA+	25	1010	0.107	27.013	Random	0.759	0.722‒0.792	0.00
SMA+	16	720	0.000	84.593	Random	0.476	0.365‒0.589	0.46
AMA+	5	405	0.000	80.700	Random	0.105	0.048‒0.214	0.92
LKM1	11	547	0.408	3.595	Random	0.070	0.049‒0.100	0.04
**AS-AIH**								
ANA+	7	358	0.028	57.609	Random	0.720	0.604‒0.812	0.76
SMA+	4	313	0.714	0.000	Random	0.374	0.322‒0.429	0.49
AMA+	2	56	0.780	0.000	Random	0.054	0.018‒0.155	-
LKM1	3	80	0.812	0.000	Random	0.027	0.007‒0.102	0.18
**ALF-AIH**								
ANA	7	145	0.001	73.781	Random	0.857	0.642‒0.953	0.00
SMA	4	116	0.096	52.797	Random	0.302	0.162‒0.493	0.44
AMA	3	102	0.324	11.265	Random	0.106	0.046‒0.226	0.03
LKM1	3	102	0.993	0.000	Random	0.029	0.009‒0.086	0.36

### Outcomes

The overall mortality rate was 12.7% (95% CI: 0.088‒0.178), with an overall transplantation rate of 15.4% (95% CI: 0.079‒0.279), a relapse rate of 24.4% (95% CI: 0.114‒0.449), and a graft-free survival rate of 45.8% (95% CI: 0.215‒0.722) (Table 6).

**Table 6:** Outcomes related to acute-onset AIH.

	Studies (n)	Participants (n)	Heterogeneity			Meta analysis		Egger
			p	*I*^2^	Model	Pooled proportion	95% CI	
**ALL**								
Mortality	17	877	0.001	59.859	Random	0.127	0.088‒0.178	0.42
Liver transplantation	13	784	0.000	89.821	Random	0.154	0.079‒0.279	0.13
Recurrence	5	170	0.004	73.646	Random	0.244	0.114‒0.449	0.82
Survived without liver transplantation	4	353	0.000	92.311	Random	0.458	0.215‒0.722	0.52
**A-AIH**								
Mortality	10	415	0.237	22.351	Random	0.089	0.057‒0.137	0.43
Liver transplantation	7	327	0.026	58.098	Random	0.045	0.017‒0.116	0.36
Recurrence	4	137	0.015	71.154	Random	0.302	0.139‒0.538	0.91
Treat with corticosteroid	8	337	0.193	29.420	Random	0.930	0.872‒0.963	0.08
Clinical remission	5	174	0.002	76.372	Random	0.814	0.583‒0.932	0.047
**AS-AIH**								
Mortality	5	340	0.305	17.151	Random	0.138	0.090‒0.205	0.14
Liver transplantation	4	333	0.000	85.488	Random	0.257	0.107‒0.501	0.89
Clinical remission	3	253	0.141	48.869	Random	0.659	0.538‒0.762	0.067
**ALF-AIH**								
Mortality	4	41	0.047	62.285	Random	0.412	0.165‒0.712	0.93
Liver transplantation	3	96	0.778	0.000	Random	0.593	0.492‒0.687	0.80

Mortality rates gradually increased with disease severity: 8.9% (95% CI: 0.057‒0.137) for A-AIH, 13.8% (95% CI: 0.090‒0.205) for AS-AIH, and 41.2% (95% CI: 0.165‒0.712) for ALF-AIH. There was a statistically significant difference between the A-AIH and ALF-AIH groups. The transplantation rate similarly escalated with severity, ranging from 4.5% in A-AIH, 25.7% in AS-AIH, to 59.3% in ALF-AIH. Once again, the statistical difference was only significant between the A-AIH and ALF-AIH groups (Table 6).

The relapse rate in the A-AIH group stood at 30.2%. However, the number of studies in the remaining two groups was fewer than two, precluding any combination of results. Steroid use was the primary treatment, implemented in 93% of cases (95% CI: 0.872‒0.963). The clinical remission rate was 81.4% (95% CI: 0.583‒0.932) in the A-AIH group and 65.9% (95% CI: 0.538‒0.762) in the AS-AIH group. Yet, no statistically significant difference emerged between the two groups (Table 6).

### Histological examination

Owing to the insufficient number of AS-AIH and ALF-AIH studies, the pathology was primarily derived from the A-AIH group. The most prevalent pathological findings included lobulitis in 91.2% of cases, plasma cell infiltration in 88.2%, and interface hepatitis in 85.3%. Rosettes, a feature typical of AIH, were present in 52.4% of A-AIH cases (95% CI: 0.353‒0.689), and in 28.9% of AS-AIH cases (95% CI: 0.150‒0.485), which was not statistically different between the two groups. Uncommon in classical AIH, bile duct injury was observed in biopsy samples of about 43% of acute AIH cases (Table 7).

**Table 7:** Histological Examination of acute-onset AIH.

	Studies (n)	Participants (n)	Heterogeneity			Meta analysis		Egger
			p	*I*^2^	Model	Pooled proportion	95% CI	
**ALL**								
Interface hepatitis	10	554	0.000	76.790	Random	0.798	0.703‒0.869	0.18
Plasma cells	5	372	0.261	24.119	Random	0.871	0.822‒0.908	0.07
Rosettes	10	548	0.000	76.622	Random	0.440	0.338‒0.547	0.28
Bile duct injury	6	314	0.000	81.582	Random	0.426	0.280‒0.586	0.72
Lobulitis	5	271	0.000	87.064	Random	0.806	0.545‒0.935	0.37
Centrilobular necrosis	9	472	0.000	80.881	Random	0.514	0.376‒0.650	0.43
Inflammation grade 0	4	155	0.982	0.000	Random	0.013	0.003‒0.051	0.00
Inflammation grade 1	4	155	0.794	0.000	Random	0.048	0.023‒0.098	0.02
Inflammation grade 2	4	155	0.063	58.854	Random	0.114	0.052‒0.234	0.00
Inflammation grade 3	4	155	0.989	0.000	Random	0.697	0.620‒0.764	0.78
Inflammation grade 4	4	155	0.125	47.667	Random	0.167	0.087‒0.296	0.00
Fibrosis stage 0-1	8	350	0.000	88.952	Random	0.511	0.326‒0.693	0.99
Fibrosis stage 2	8	350	0.229	25.027	Random	0.205	0.156‒0.265	0.01
Fibrosis stage 3	8	350	0.004	66.267	Random	0.121	0.063‒0.222	0.00
Fibrosis stage 4	8	350	0.001	71.760	Random	0.166	0.085‒0.300	0.08
**A-AIH**								
Interface hepatitis	6	215	0.035	58.218	Random	0.853	0.740‒0.922	0.19
Plasma cells	4	246	0.153	43.029	Random	0.882	0.805‒0.931	0.13
Rosettes	7	223	0.000	71.154	Random	0.524	0.353‒0.689	0.79
Bile duct injury	4	144	0.002	77.950	Random	0.468	0.249‒0.700	0.72
Lobulitis	4	116	0.000	80.404	Random	0.912	0.381‒0.994	0.00
Centrilobular necrosis	7	300	0.027	77.950	Random	0.470	0.373‒0.569	0.68
Inflammation grade 0	4	155	0.982	0.000	Random	0.013	0.003‒0.051	0.00
Inflammation grade 1	4	155	0.794	0.000	Random	0.048	0.023‒0.098	0.02
Inflammation grade 2	4	155	0.063	58.854	Random	0.114	0.052‒0.234	0.00
Inflammation grade 3	4	155	0.989	0.000	Random	0.697	0.620‒0.764	0.78
Inflammation grade 4	4	155	0.125	47.667	Random	0.167	0.087‒0.296	0.00
Fibrosis stage 0-1	8	350	0.000	88.952	Random	0.511	0.326‒0.693	0.99
Fibrosis stage 2	8	350	0.229	25.027	Random	0.205	0.156‒0.265	0.01
Fibrosis stage 3	8	350	0.004	66.267	Random	0.121	0.063‒0.222	0.00
Fibrosis stage 4	8	350	0.001	71.760	Random	0.166	0.085‒0.300	0.08
**AS-AIH**								
Interface hepatitis	2	177	0.059	71.981	Random	0.803	0.509‒0.941	‒
Rosettes	2	177	0.117	59.363	Random	0.289	0.150‒0.485	‒

Compared to the inflammatory grade of A-AIH, which primarily clustered in Grade 3 (69.7%) and Grade 4 (16.7%), fibrosis was predominantly found in F0-1 (51.1% with 95% CI: 0.326‒0.693). This was significantly higher than in F2 (20.5% with 95% CI: 0.156‒0.265), F3 (12.1% with 95% CI: 0.063‒0.222), and F4 (16.6% with 95% CI: 0.085‒0.300) (Table 7).

### Acute-onset Autoimmune Hepatitis (A-AIH) and Chronic Autoimmune Hepatitis (C-AIH)

#### Characteristics

Compared to typical Chronic AIH (C-AIH), patients with acute-onset AIH (A-AIH) tended to be younger (MD = -0.253, 95% CI: -0.449 to -0.057). However, there was no significant difference in the ratio of female patients between the two groups (Table 8).

**Table 8:** Clinical features of A-AIH compared with C-AIH.

	Studies (n)	Participants (n)	Heterogeneity			Meta analysis		Egger
			p	*I*^2^	Model	Mean difference/ Odds ratio	95% CI	
**Characteristics**								
Age	8	438	0.720	0.000	Random	-0.253	-0.449‒-0.057	0.35
Sex (females)	11	1133	0.983	0.000	Random	1.215	0.888‒1.663	0.88
**Symptom**								
Jaundice	5	300	0.443	0.000	Random	5.841	3.446‒9.899	0.85
Fatigue	3	244	0.998	0.000	Random	1.320	0.786‒2.216	0.73
Abdominal pain	3	244	0.837	0.000	Random	0.698	0.331‒1.474	0.07
Anorexia	3	244	0.851	0.000	Random	7.323	4.014‒13.359	0.47
Ascites	4	270	0.891	0.000	Random	1.153	0.652‒2.039	0.12
Weight loss	3	244	0.449	0.000	Random	1.543	0.739‒3.221	0.98
Yellow urine	3	244	0.815	0.000	Random	4.290	2.325‒7.826	0.68
Lower limb swelling	3	244	0.988	0.000	Random	0.464	0.189‒1.138	0.32
Extrahepatic autoimmune disease	4	825	0.503	0.000	Random	0.630	0.455‒0.874	0.41
**Laboratory examination**								
ALB (g/L)	6	289	0.000	87.778	Random	-0.479	-1.231‒0.272	0.79
GLB (g/L)	4	225	0.189	37.200	Random	-0.457	-0.831‒‒0.084	0.83
TB (mg/dL)	7	271	0.000	80.790	Random	1.607	0.932‒2.281	0.03
AST (IU/L)	6	306	0.003	72.162	Random	1.920	1.368‒2.472	0.04
ALT (IU/L)	6	305	0.000	89.885	Random	2.032	1.081‒2.983	0.12
**Autoantibody**								
ANA+	12	1222	0.001	64.025	Random	1.706	0.960‒3.033	0.23
SMA+	8	962	0.652	0.000	Random	1.243	0.939‒1.647	0.61
LKM1	6	889	0.943	0.000	Random	0.854	0.505‒1.445	0.90
**Outcomes**								
Mortality	4	174	0.550	0.000	Random	7.141	1.569‒32.506	0.21
Liver transplantation	3	148	0.927	0.000	Random	4.994	1.108‒22.504	0.05
**Histological examination**								
Rosettes	4	331	0.140	45.199	Random	3.629	1.500‒8.780	0.10
Interface hepatitis	3	135	0.743	0.000	Random	1.775	0.691‒4.563	0.35
Bile duct injury	2	110	0.001	91.516	Random	2.918	1.161‒7.331	‒
Centrilobular necrosis	3	140	0.019	74.708	Random	1.691	0.326‒8.771	0.75
Inflammation grade 0	3	242	0.946	0.000	Random	0.417	0.067‒2.605	0.01
Inflammation grade 1	3	242	0.977	0.000	Random	0.101	0.030‒0.340	0.00
Inflammation grade 2	3	242	0.993	0.000	Random	0.141	0.061‒0.326	0.60
Inflammation grade 3	3	242	0.940	0.000	Random	3.594	2.076‒6.224	0.16
Inflammation grade 4	3	242	0.986	0.000	Random	25.900	4.816‒139.276	0.06
Fibrosis stage 0‒1	4	272	0.650	0.000	Random	0.746	0.446‒1.248	0.55
Fibrosis stage 2	4	272	0.722	0.000	Random	0.630	0.315‒1.258	0.58
Fibrosis stage 3	4	272	0.163	41.456	Random	1.553	0.595‒4.055	0.64
Fibrosis stage 4	4	272	0.944	0.000	Random	1.438	0.832‒2.486	0.00

#### Symptoms

When compared to C-AIH, anorexia (OR = 7.323, 95% CI: 4.014‒13.359) was the most prominent symptom in A-AIH patients. Jaundice and yellow urine followed, with no significant difference noted among the remaining symptoms. Moreover, the incidence of A-AIH combined with autoimmune disease (OR = 0.63, 95% CI: 0.455‒0.874) was significantly lower than in C-AIH. Notably, unlike the high heterogeneity of symptoms across severity levels, these studies exhibited very low heterogeneity (*I*^2^ = 0) (Table 8).

#### Laboratory examination

Patients with A-AIH demonstrated significantly lower Globulin (GLB) levels (MD = -0.457, 95% CI: -0.831 to -0.084) compared to those with C-AIH. In contrast, Total Bilirubin (TB) (MD = 1.607, 95% CI: 0.932‒2.281), Aspartate Aminotransferase (AST) (MD = 1.920, 95% CI: 1.368‒2.472), and Alanine Aminotransferase (ALT) (MD = 2.032, 95% CI: 1.081‒2.983) levels were significantly higher than in patients with C-AIH. There was, however, no statistically significant difference in ANA, SMA, and LKM1 positivity between the two groups (Table 8).

#### Outcomes

The mortality (OR = 7.141, 95% CI: 1.569‒32.506) and transplantation rates (OR = 4.994, 95% CI: 1.108‒22.504) were significantly higher in patients with A-AIH than in those with C-AIH (Table 8).

#### Histological examination

Although A-AIH patients often lacked rosettes, combined results showed the presence of rosettes in A-AIH (OR = 3.629, 95% CI: 1.500‒8.780) was significantly higher than in C-AIH patients (Table 8). Consistent with the previous results, A-AIH showed significantly more bile duct injuries than C-AIH (OR = 2.918, 95% CI: 1.161‒7.331) (Table 8).

By comparison, patients with A-AIH had higher inflammation grades of 3 (OR = 3.594, 95% CI: 2.076‒6.224) and 4 (OR = 25.900, 95% CI: 4.816‒139.276), while C-AIH patients typically had milder inflammation grades of 1 (OR = 0.101, 95% CI: 0.030‒0.340) and 2 (OR = 0.141, 95% CI: 0.061‒0.326) (Table 8).

The degree of fibrosis in A-AIH and C-AIH patients showed slight variation, though these differences were not statistically significant.

#### Publication bias

Egger's test was used for each outcome, and the results are presented in the last column of each table, and the majority of significant results were free of publication bias.

## Discussion

The notion of acute Autoimmune Hepatitis (AIH) was first introduced by Lefkowitch et al. in 1984, who detailed two instances of AIH displaying histological indications of acute hepatitis.^43^ Acute-onset AIH is characterized by its swift progression and potential for misdiagnosis due to its resemblance to other illnesses causing liver dysfunction. Therefore, comprehending the clinical features of Acute-onset AIH is crucial for its accurate diagnosis and effective treatment.

Our study showed that patients with A-AIH were younger compared to patients with C-AIH. Although no continuous statistical differences were found in demographic characteristics between different severities of acute-AIH, there was indeed a tendency for the female sex ratio to gradually decrease as the severity of acute AIH worsened, eventually showing a significant difference between A-AIH and C-AIH. It is well-documented that females have a considerably higher incidence of certain autoimmune diseases, such as Graves' disease or scleroderma, possibly due to differences in immune function between sexes.^44^ Genetic factors, sex hormones, and other influences could enhance women's immune response, yet simultaneously increase their susceptibility to autoimmune diseases. Historically, women spent most of their lives in states of pregnancy and lactation, providing long-term regulation of the immune system.^44^ However, modern contraception and fewer pregnancies have potentially disrupted this immune regulation. One study indicated that women with autoimmune hepatitis initiated oral contraception at an earlier age and more frequently, with fewer lifetime pregnancies compared to the control group.^45^ Our findings indicate a decrease from 81% to 65% in female prevalence from acute non-severe exacerbation to fulminant hepatic failure in AIH. If patients with an acute onset of chronic AIH could be further excluded, there is a possibility that the male-to-female ratio may even out to 1:1. This suggests that severe A-AIH differs substantially from typical Chronic AIH (C-AIH), and that the fulminant progression may bypass the influence of gender, notably the predominant female impact. Considering the female predominance in autoimmune hepatitis patients, it is imperative to further investigate the role of hormones in both acute and chronic AIH, which may reveal the underlying causes of variation in the course and progression of AIH.

AIH has been demonstrated to be strongly correlated with extra-hepatic autoimmune diseases.^46^ As specified in the 1999 International Autoimmune Hepatitis Group (IAIHG) criteria,^40^ two points are added to the diagnosis score for coexisting immune disorders. This correlation is primarily due to shared genetic backgrounds, susceptibility genes, and other contributing factors. However, the mechanisms underlying this intricate association are still not extensively understood. Shen et al.^37^ discovered that about 22% of acute AIH cases were accompanied by an extrahepatic autoimmune disease, a figure slightly higher than the 19% seen in classical AIH. Notably, the difference between these two was not statistically significant. Conversely, our study revealed a notably lower co-occurrence of extrahepatic autoimmune diseases in acute AIH (A-AIH) compared to Chronic AIH (C-AIH).

The clinical implications and prognostic influence of extrahepatic autoimmune diseases on AIH remain a contentious subject. Some researchers suggest that AIH patients with concurrent extrahepatic autoimmune diseases are mostly asymptomatic at presentation and exhibit less severe disease progression.^46^ It's also been suggested that the co-occurrence of extrahepatic autoimmune liver diseases does not significantly impact AIH's progression, prognosis, or survival.^47^ However, this contrasts with findings by Birn-Rydder et al., who reported increased mortality in AIH patients with extrahepatic autoimmune diseases.^48^

Our results show that A-AIH is significantly less likely to be associated with other autoimmune diseases than C-AIH. We observed a trend of declining comorbidity as the severity increased, i.e., 23% of A-AIH, 21% of Acute-Severe AIH (AS-AIH), and 7% of Acute Liver Failure AIH (ALF-AIH) cases^4^ were accompanied by extrahepatic autoimmune diseases, though the differences were not statistically significant. This pattern seems to bolster the notion that the presence of other autoimmune diseases may serve as a protective factor for AIH patients.^46^

The co-occurrence of other autoimmune diseases with AIH suggests a potential linkage between these diseases and the prognosis of A-AIH, given the significantly different outcomes of AIH with acute manifestations of varying severity. We postulate that this could be due to the influence of extrahepatic autoimmune diseases on immune homeostasis, although the mechanism warrants further exploration. It's important to note that although AIH often coexists with certain immune disorders, this relationship isn't always bidirectional. While 0.7%‒18.8% of AIH cases may be associated with Systemic Lupus Erythematosus (SLE), up to 60% of patients with SLE may develop hepatic insufficiency, yet rarely coexist with autoimmune hepatitis.^49^

Recent advancements in molecular genetics, including Genome-Wide Association Studies (GWAS) and risk-associated Single Nucleotide Polymorphism (SNP) research, have greatly improved our understanding of autoimmune diseases' coexistence. These findings suggest that a shared genetic background contributes to the concurrent presence of autoimmune liver disease and other extrahepatic autoimmune diseases.^50^

The primary challenge in managing acute-onset AIH is timely and accurate diagnosis, as untreated cases can swiftly progress to liver failure or even result in death. We found that over half of patients might exhibit symptoms like jaundice (74.5%), anorexia (62.0%), and fatigue (59.9%), all of which could stem from elevated bilirubin levels. Subgroup analysis revealed that while fatigue and anorexia are common nonspecific symptoms of chronic autoimmune hepatitis, anorexia was the most prominent symptom in patients with Acute AIH (A-AIH) compared to those with Chronic AIH (C-AIH). This suggests that patients with gastrointestinal symptoms such as anorexia warrant closer monitoring.

Acute-onset AIH typically lacks the clinical features characteristic of chronic autoimmune hepatitis, making the identification of serological features even more crucial. Our results indicated that White Blood Cell (WBC) and Platelet (PLT) counts were within the normal range. However, it's important to note that these factors can serve as predictors for complications like infections and treatment outcomes, and thus require dynamic observation for meaningful interpretation. A study by Shen et al.^37^ reported lower Albumin (ALB) levels in the acute group, suggesting that a deteriorating liver condition in A-AIH affects ALB production. In line with this, Kessler et al.^5^ proposed that ALB might play a role in the acute phase response, hence hypoalbuminemia is a suitable indicator of A-AIH. Yet, our results showed no significant difference in ALB levels between A-AIH and C-AIH, possibly due to the high heterogeneity of the included studies (*I*^2^ = 87.778). Unfortunately, the Acute-Severe AIH (AS-AIH) and Acute Liver Failure AIH (ALF-AIH) groups lacked sufficient studies to further examine the role of ALB.

Acute-onset AIH is primarily defined as acute exacerbation symptoms accompanied by elevated serum bilirubin and/or serum alanine aminotransferase.^38^ Our results affirmed that this accurately reflects the characteristics of acute exacerbation of autoimmune hepatitis. Although it is reliable as an indicator of acute onset of disease, but it's the bilirubin and coagulation indices that can further differentiate the degree of severity. Aminotransferases do not serve such a role. Despite many unresolved questions in diagnosing acute manifestations of AIH, current indicators of response severity perform adequately.

Autoantibodies are a crucial diagnostic criterion in all autoimmune hepatitis scoring systems, serving significant roles in diagnosis and subtype determination.^2^ A distinguishing feature of A-AIH is often the absence of typical ANA, ASMA, and LKM-1. However, this feature was not significant in our meta-analysis and was found to be independent of severity. This might be due to a significant proportion of Acute-onset Chronic AIH patients mixed into this group. The value of autoantibodies in acute-onset AIH remains uncertain, but with current results, autoantibodies still remain key diagnostic indicators for both true A-AIH and acute onset of C-AIH.

In Furthermore, Globulin (GLB) levels were significantly lower in A-AIH than in C-AIH, and also some researchers have suggested that globulin may be an important indicator for differentiating between A-AIH and AC-AIH.^37^ An Italian study^7^ revealed that histologically identified acute onset AIH (between true A-AIH and AC-AIH) had lower GLB (19 vs. 24, p = 0.005) and IgG (1.12 vs. 1.4, p = 0.03) levels, which might explain why we didn't find a difference across different degrees of severity.

Pathologically, acute AIH is predominantly characterized by acute lobulitis with centrilobular necrosis, although the intrinsic significance of this still requires further verification. Our findings in the A-AIH group align with this, with lobulitis accounting for 91.2% and centrilobular necrosis 47%. Notably, acute AIH, often thought to lack typical pathology such as interface hepatitis and rosettes, was still present in half or even over 80% of cases. The presence of rosettes was significantly higher in A-AIH than in C-AIH. This may be because, along with true A-AIH, there is also some acute onset of C-AIH in a sub-clinical stage. A recent study^39^ showed that 16.7% of A-AIH patients and 37.5% of AC-AIH patients presented with rosettes, along with a similar profile in interface hepatitis, suggesting that many previous studies have confused A-AIH and AC-AIH.

Regarding the degree of fibrosis, nearly half of the patients were in stages F2-F4, indicating that at least half of the patients in the current study were experiencing an acute exacerbation of C-AIH. Future studies should more effectively differentiate between A-AIH and AC-AIH. More accurate subgroup analyses may yield different results.^37^

Bile duct injuries, although uncommon in classical AIH, have shown that Acute-Severe AIH (AS-AIH) slightly less frequently present with bile duct injuries than C-AIH.^36^ Our results, however, found that bile duct injuries were more common in A-AIH, significantly differing from C-AIH. Bile duct injury appears to be one of the histologic criteria to distinguish between acute and chronic stages. Yet, due to the small number of included studies and the high heterogeneity (*I*^2^ = 91.516), more research is needed to understand the significance of bile duct injury on the diagnosis and prognosis of A-AIH.

Our meta-analysis comes with certain limitations. Parts of the data types and formats were not compatible for results integration, leading us to discard some significant findings. Moreover, only a few studies provided a comprehensive documentation of the disease's full course, and incomplete data made further analysis challenging. Acute-onset AIH, being a manifestation of rare diseases, made it difficult for us to gather sufficient data to classify by ethnicity, region, and age, thereby increasing the heterogeneity of our data. Most importantly, not all studies have been able to discern AC-AIH due to diagnostic complexities and difficulties in differential diagnosis of A-AIH. While it has been posited^37^ that initial differentiation between acute manifestations and chronic autoimmune hepatitis, based solely on serologic markers without using acute and chronic histologic features, is reliable, a more granular classification might reveal different results or even alter the current understanding of acute-onset AIH.

## Conclusion

In conclusion, this is the first systematic summary of acute-onset AIH. Our study found that patients with acute-onset AIH were younger and that the gender difference narrowed with increasing disease severity. Among numerous nonspecific symptoms, apart from jaundice, anorexia was the symptom that differed most from C-AIH. The presence of comorbid extrahepatic autoimmune diseases correlated with less severe disease in acute-onset AIH, implying a potential protective role. Current laboratory tests for identifying acute-onset AIH and differentiating its severity demonstrated moderately acceptable performance. Both autoantibody and histologic manifestations of A-AIH seem to be affected by AC-AIH, rendering some of the typically rare manifestations of A-AIH statistically indistinguishable from those of standard AIH. The current evidence supports only our initial conclusions, and future studies need to underscore the importance of comprehensive documentation and follow-up.

## Ethics approval and consent to participate

Since this is a meta-analysis, which involves the synthesis of data from previously published studies, no new data were collected directly from human participants. Therefore, formal ethics approval from an Institutional Review Board (IRB) was not required. All the studies included in this meta-analysis had already obtained appropriate ethics approvals from their respective institutions at the time of original data collection. We have carefully reviewed the ethics statements in each of the included studies to ensure that the data used in our analysis were collected in accordance with ethical principles. As no new participants were involved in this study, the issue of consent to participate did not arise. However, we respect the privacy and rights of all individuals whose data are included in the original studies. We have made every effort to ensure that the data extraction and analysis were conducted in a way that maintains the confidentiality and integrity of the original data sources.

### Consent for publication

Not applicable.

### Availability of data and material

The data for this meta-analysis were retrieved from well-known databases like PubMed and Embase. All data are available within the text. The search strategies are detailed in the Methods section. All data are from published studies and can be accessed via the original journals' websites. For paywalled articles, institutional subscriptions or requests to authors may help. We've compiled an analyzed dataset. Upon request, the corresponding author can provide this dataset and relevant analysis code (if any), subject to the original data source terms. Also, the data extraction form and study metadata are available to ensure transparency and reproducibility.

### Clinical trial number

Not applicable.

### Funding

This work was supported by Sponsored Programs: Xijing Discipline Boosting Project of Xijing Hospital (no. XJZT21CZ02).

## Declaration of competing interest

The authors declare no conflicts of interest.
