# Supplementary material for: Clinical features of acute-onset autoimmune hepatitis: a systematic review and meta-analysis
Source: Clinics (Sao Paulo). 2026 Sep 24;81:101118. doi: 10.1016/j.clinsp.2026.101118 (PMC13634725; doi:10.1016/j.clinsp.2026.101118)
Supplement: Supplementary file 1 [file mmc1.docx]

CLINICS-D-25-01167_Supplementary Material

**Supplementary Figure 1** PRISMA flow chart of selection process to identify eligible studies

**Supplementary Material**

**Supplementary Material 1** Risk of bias summary.

| **Study** | **Selection** | | | | **Cmparability** | **Exposure** | | | **Scores** |
| --- | --- | --- | --- | --- | --- | --- | --- | --- | --- |
|  | **Adequate definition of cases** | **Representativeness of the cases** | **Selection of controls** | **Definition of controls** | **Control for important factor** | **Ascertainment of exposure** | **Same method of ascertainment for cases and controls** | **Non-response rate** |  |
| Urzúa Á 2022 | ★ | ★ | ★ | ★ | ★ | ‒ | ★ | ‒ | 6 |
| Y Han 2022 | ★ | ★ | ★ | ★ | ★★ | ★ | ★ | ‒ | 8 |
| Abdulrahman A Aljumah 2019 | ★ | ★ | ‒ | ‒ | ★★ | ★ | ★ | ‒ | 6 |
| Yi Shen 2018 | ★ | ★ | ★ | ★ | ★★ | ★ | ★ | ‒ | 8 |
| Hong Nie 2021 | ★ | ★ | ★ | ★ | ★ | ★ | ★ | ‒ | 7 |
| Jia Xia 2021 | ★ | ★ | ★ | ★ | ★★ | ★ | ★ | ‒ | 8 |
| William R Kessler 2004 | ★ | ★ | ★ | ★ | ★★ | ★ | ★ | ‒ | 8 |
| Satoru Joshita 2018 | ★ | ★ | ★ | ★ | ★★ | ★ | ★ | ★ | 9 |
| Paolo Muratori 2018 | ★ | ★ | ★ | ★ | ★★ | ★ | ★ | ★ | 9 |
| Keiichi Fujiwara 2018 | ★ | ★ | ★ | ★ | ★ | ★ | ★ | ★ | 8 |
| Kazuhide Yamamoto 2013 | ★ | ★ | ‒ | ‒ | ★ | ★ | - | ★ | 5 |
| Y Miyake 2010 | ★ | ★ | ★ | ★ | ★★ | ★ | ★ | - | 8 |
| Keiichi Fujiwara 2016 | ★ | ★ | ★ | ★ | ★★ | ★ | ★ | - | 8 |
| R Ferrari 2004 | ★ | ★ | ★ | ★ | ★★ | ★ | ★ | ★ | 9 |
| Masaki Iwai 2008 | ★ | ★ | ★ | ★ | ★★ | ★ | ★ | ‒ | 8 |
| Vratislav Smolka 2020 | ★ | ★ | ★ | ★ | ★ | ★ | ★ | ‒ | 7 |
| Hiep Nguyen Canh 2017 | ★ | ★ | ‒ | ‒ | ★ | ★ | ★ | ‒ | 5 |
| G A Nikias 1994 | ★ | - | ★ | ★ | ★ | ★ | ★ | ‒ | 6 |
| Keiichi Fujiwara 2011.04 | ★ | ★ | ★ | ★ | ★★ | ★ | ★ | ‒ | 8 |
| Luis Téllez et al. 2022 | ★ | ★ | ‒ | ‒ | ★ | ★ | ★ | ★ | 6 |
| Shin Yasui 2011.03 | ★ | ★ | ★ | ★ | ★★ | ★ | ★ | ‒ | 8 |
| Nikhil Sonthalia 2017 | ★ | ★ | ★ | ★ | ★ | ★ | ★ | ‒ | 7 |
| Norberto C Chavez-Tapia 2007 | ★ | ★ | ‒ | ‒ | ★ | ★ | ★ | ‒ | 5 |
| Kazufumi Dohmen 2017 | ★ | ★ | ★ | ★ | ★ | ★ | ★ | ‒ | 7 |
| Andrew D Yeoman 2014 | ★ | ★ | ★ | ★ | ★ | ★ | ★ | ‒ | 7 |
| Mafalda João 2022 | ★ | ★ | ‒ | ‒ | ★ | ★ | ★ | ‒ | 5 |
| Keiichi Fujiwara 2017 | ★ | ★ | ‒ | ‒ | ★ | ★ | ★ | ‒ | 5 |
| Keiichi Fujiwara 2011.03 | ★ | ★ | ★ | ★ | ★★ | ★ | ★ | ‒ | 8 |
| A Di Giorgio 2015 | ★ | ★ | ★ | ★ | ★ | ★ | ★ | ‒ | 7 |
| Shin Yasui 2011.06 | ★ | ★ | ★ | ★ | ★ | ★ | ★ | ‒ | 7 |
| R Todd Stravitz 2011 | ★ | ★ | ‒ | ‒ | ★ | ★ | ★ | ‒ | 5 |
| Burgart.1995 | ★ | ‒ | ‒ | ‒ | ★ | ★ | ★ | ‒ | 4 |
| Masanori Abe 2007 | ★ | ‒ | ★ | ★ | ★ | ★ | ★ | ‒ | 6 |
| Masanori Abe 2001 | ★ | ★ | ★ | ★ | ★ | ★ | ★ | ‒ | 7 |
| HuanRong Hou 2016 | ★ | ★ | ‒ | ‒ | ★ | ★ | ‒ | ‒ | 4 |
| XiaoQing Fu 2008 | ★ | ★ | ★ | ★ | ★ | ★ | ★ | ‒ | 7 |

**Supplementary Table 1** Basic summary of the literature included.

| **Author** | **Country** | **Year** | **Type of study** | **Patients’ characteristics** | **Participants (male/ female)** | **Median/ Mean age (IQR/SD)** | **Quality score** |
| --- | --- | --- | --- | --- | --- | --- | --- |
| Urzúa Á et al 2022 | Chile | 2000‒2008 | Retrospective study | A-AIH | 126 (29/97) | 49.0±15.0 | 6 |
| Y Han 2022 | China | 2008‒2019 | Retrospective study | AS-AIH/C-AIH | 41 (2/39) | 54.2±10.6 | 8 |
| Abdulrahman A Aljumah 2019 | Saudi Arabia | 1999.01‒2017.12 | Retrospective cohort study | A-AIH | 70 (29/41) | 33.8±1.5 | 6 |
| Yi Shen 2018 | China | 2013.10‒2016.11 | Retrospective study | A-AIH/C-AIH | 80 (13/67) | 52.0±12.0 | 8 |
| Jia Xia 2021 | China | 2015.01‒2017.01 | Retrospective study | A-AIH/C-AIH | 37 (6/31) | 53.12 ± 13.06 | 7 |
| Hong Nie 2021 | China | 2010.5‒2020.11 | Retrospective study | A-AIH/C-AIH | 28 (4/24) | 53.4±11.3 | 8 |
| William R Kessler 2004 | US | 1993‒2002 | Retrospective study | A-AIH/C-AIH | 30 | 40.0±15.9 | 8 |
| Satoru Joshita 2018 | Japan | 2005‒2014 | Retrospective study | A-AIH | 86 (11/75) | 55.0 | 9 |
| Paolo Muratori 2018 | Italiy | 2003‒2020 | Retrospective study | A-AIH/AC-AIH | 479 (204/275) | 42.0 | 9 |
| Keiichi Fujiwara 2018 | Japan | 2001‒2015 | Retrospective study | A-AIH | 30 (9/21) | 55.0±13.1 | 8 |
| Kazuhide Yamamoto 2013 | Japan | 2007.01‒2008.12 | Retrospective study | A-AIH | 96 (18/78) | 58.0 | 5 |
| Y Miyake 2010 | Japan | 1989.03‒2008.08 | Retrospective study | A-AIH | 176 (53/123) | 55.0 | 8 |
| Keiichi Fujiwara 2016 | Japan | 2001‒2015 | Retrospective study | A-AIH | 46 (11/35) | 55.9±14.2 | 8 |
| R Ferrari 2004 | Italiy | 2004 | Prospective observational study | A-AIH | 22 (4/18) | 39.5±20.2 | 9 |
| Masaki Iwai 2008 | Japan | 1999‒2008 | Retrospective study | A-AIH/C-AIH | 29 (6/23) | 44.8±21.9 | 8 |
| Vratislav Smolka 2020 | Czech | 2000‒2018 | Retrospective study | A-AIH/C-AIH | 40 (14/26) |  | 7 |
| Hiep Nguyen Canh 2017 | Japan | 2005‒2014 | Retrospective study | A-AIH | 87 (12/75) | 54.5±15.3 | 5 |
| G A Nikias.1994 | US | 1967‒1994 | Retrospective study | A-AIH/C-AIH | 26 (6/20) | 44.0±4.0 | 6 |
| Keiichi Fujiwara 2011.08 | Japan | 2000‒2009 | Retrospective study | A-AIH | 55 (12/43) | 51.6 ± 14.3 | 8 |
| Luis Téllez et al. 2022 | spain | 2002.01‒2019.01 | Retrospective cohort study | AS-AIH | 242 (180/62) | 49.6±16.8 | 6 |
| Shin Yasui 2011.03 | Japan | 1990‒2009 | Retrospective study | AS-AIH | 28 (7/21) | 46.9±15.8 | 8 |
| Nikhil Sonthalia 2017 | India | 2011.01‒2015.12 | Retrospective study | AS-AIH | 101 (10/91) |  | 7 |
| Norberto C Chavez-Tapia 2007 | Mexico | 1984‒2004 | Retrospective study | ALF-AIH | 11 (2/9) | 33.0 | 5 |
| Kazufumi Dohmen 2017 | Japan | 2008.01‒2016.01 | Retrospective study | A-AIH/C-AIH | 32 (4/28) | 56.9±14.6 | 7 |
| Andrew D Yeoman 2014 | UK | 1999‒2009 | Retrospective study | AS-AIH | 32 (9/23) |  | 7 |
| Mafalda João 2022 | Portugal | 2016‒2020 | Retrospective cohort study | AS-AIH | 26 (4/22) | 56(45-69) | 5 |
| Keiichi Fujiwara 2017 | Japan | 1990‒2009 | Retrospective study | AS-AIH | 23 (7/16) | 48.8 ± 15.4 | 5 |
| Keiichi Fujiwara 2011.04 | Japan |  | Retrospective study | A-AIH/AS‐AIH /ALF‐AIH | 27 (4/23) | 51.2±15.4 | 8 |
| A Di Giorgio 2015 | Italiy | 1996‒2012 | Retrospective study | ALF-AIH | 1 0(3/7) |  | 7 |
| Shin Yasui 2011.06 | Japan | 2000‒2009 | Retrospective study | A-AIH | 17 (2/15) | 54.6±13.6 | 7 |
| R Todd Stravitz 2011 | US | 1998‒2008 | Retrospective study | ALF-AIH | 72 (30/42) | 41.0±1.7 | 5 |
| Burgart 1995 | US | 1967‒1994 | Retrospective study | A-AIH | 26 (5/21) | 50.0±3.0 | 4 |
| Masanori Abe 2007 | Japan |  | Retrospective study | A-AIH/C-AIH | 77 |  | 6 |
| Masanori Abe 2001 | Japan |  | Retrospective study | A-AIH/C-AIH | 13 |  | 7 |
| RongHuan Hou 2016 | China | 2009.7‒2015.4 | Retrospective study | ALF-AIH | 16 (5/11) | 56.2± 9.3 | 4 |
| XiaoQing Fu 2008 | China | 2004.1‒2006.11 | Retrospective study | A-AIH | 36 (0/36) | 49.2±11.0 | 7 |
